# Supplementary material for: Distinct and Dynamic Changes in the Temporal Profiles of Neurotransmitters in Drosophila melanogaster Brain following Volatilized Cocaine or Methamphetamine Administrations
Source: Pharmaceuticals (Basel). 2023 Oct 19;16(10):1489. doi: 10.3390/ph16101489 (PMC10609923; doi:10.3390/ph16101489)
Supplement: Supplementary file 1 [file pharmaceuticals-16-01489-s001.zip › Figure S1.pdf]

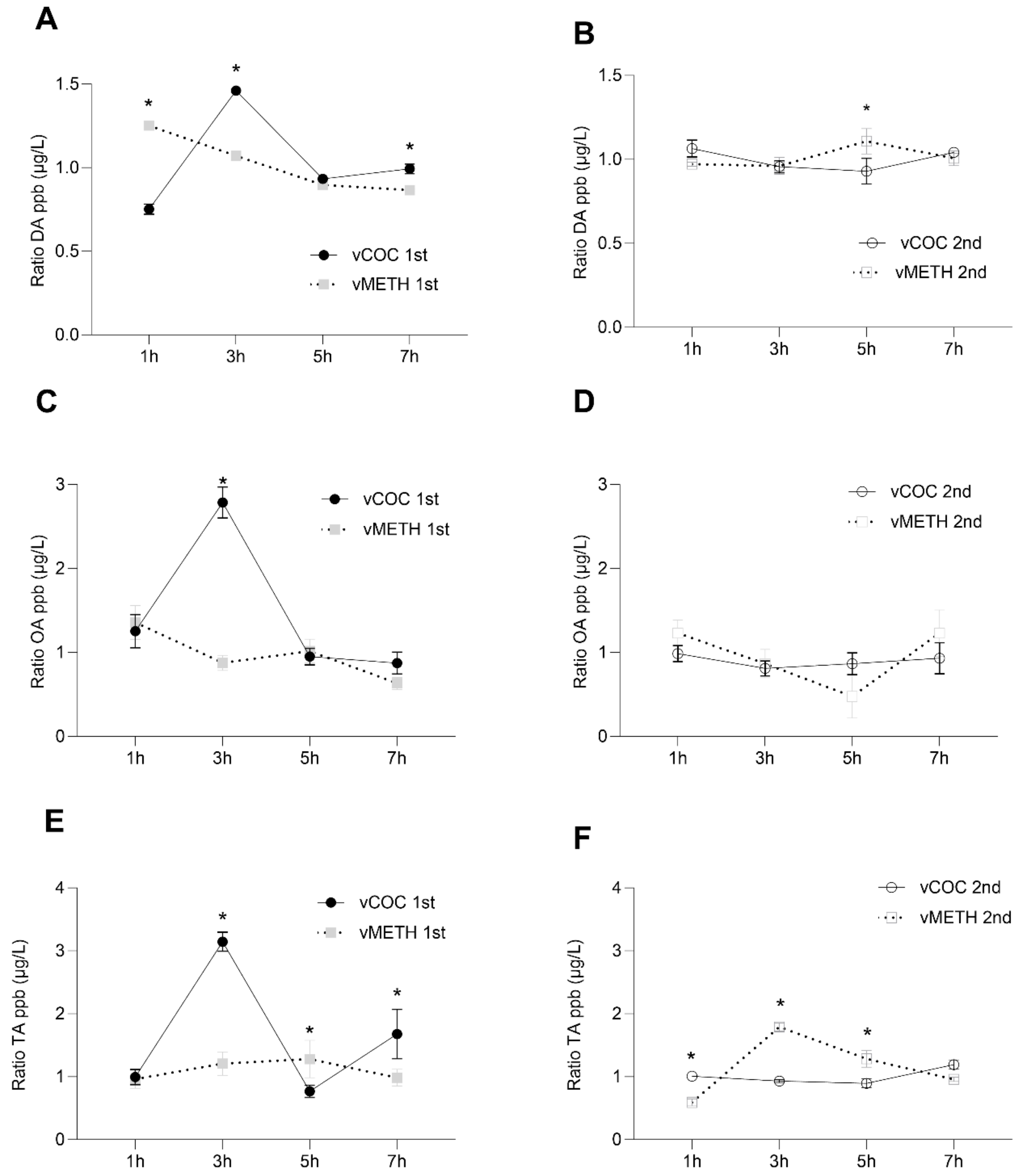

Figure S1. Temporal dynamics of changes in the concentration ratio of monoamines quantified with standards and after vCOC and vMETH administration of one and two doses. The concentration of DA, OA and TA was measured in the heads 1h, 3h, 5h and 6h after one dose of vCOC (75 µg) at 9:00, and two doses of vCOC (2 × 75 µg) administered at 9:00 and 15:00 or one (vMETH 1st) administred at 9:00 and two (vMETH 2nd) doses administered at 9:00 and 19:00. vCOC and vMETH were administered using the FlyBong method. Change between one dose of cocaine (vCOC 1st) and metamphetamine (vMETH 1st) in **A)**, dopamine (DA) **C)**, octopamine (OA) **E)** and tyramine (TA). Concentrations after exposure to two vCOC (vCOC 2nd) and two vMETH (vMETH 2nd) doses in **B)**, dopamine (DA) **D)**, octopamine (OA) **F)** and tyramine (TA). Two-way ANOVA with Tukey's multiple comparisons test. *: $p<0.05$.
