# Supplementary material for: Distinct and Dynamic Changes in the Temporal Profiles of Neurotransmitters in Drosophila melanogaster Brain following Volatilized Cocaine or Methamphetamine Administrations
Source: Pharmaceuticals (Basel). 2023 Oct 19;16(10):1489. doi: 10.3390/ph16101489 (PMC10609923; doi:10.3390/ph16101489)
Supplement: Supplementary file 1 [file pharmaceuticals-16-01489-s001.zip › Figure S2.pdf]

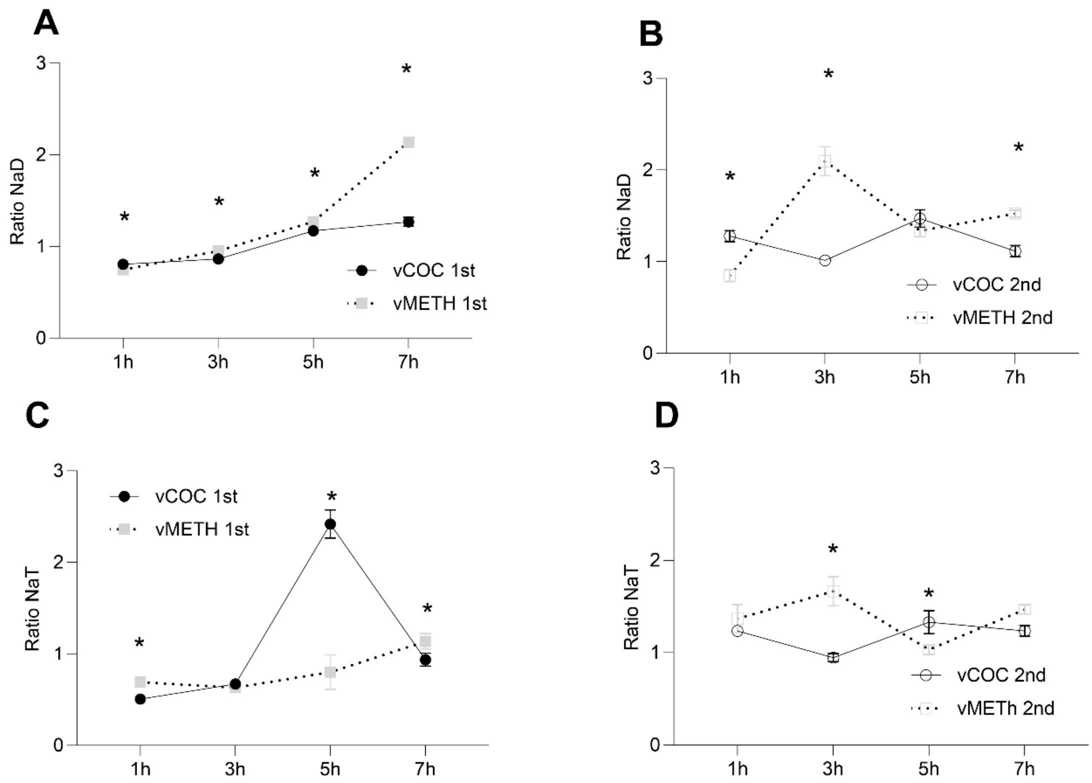

Figure S2. Temporal dynamics of changes in the concentration ratio of DA metabolite N-acetyl dopamine and TA metabolite N-acetyl tyramine semi-quantified without standards after vCOC and vMETH administration. Presentation of changes in the concentration of N-acetyl dopamine (NaD) **A)** and N-acetyl tyramine (NaT) **C)** after exposure to one (vCOC 1st) and (vMETH 1st) dose. Two-way ANOVA with Tukey's multiple comparisons test. *: $p<0.05$. Presentation of changes in the concentration of N-acetyl dopamine (NaD) **B)** and N-acetyl tyramine (NaT) **D)** after exposure to two (vCOC 2nd) and two (vMETH 2nd) doses. Two-way ANOVA with Tukey's multiple comparisons test. *: $p<0.05$.
