# Supplementary material for: Distinct and Dynamic Changes in the Temporal Profiles of Neurotransmitters in Drosophila melanogaster Brain following Volatilized Cocaine or Methamphetamine Administrations
Source: Pharmaceuticals (Basel). 2023 Oct 19;16(10):1489. doi: 10.3390/ph16101489 (PMC10609923; doi:10.3390/ph16101489)
Supplement: Supplementary file 1 [file pharmaceuticals-16-01489-s001.zip › Figure S3.pdf]

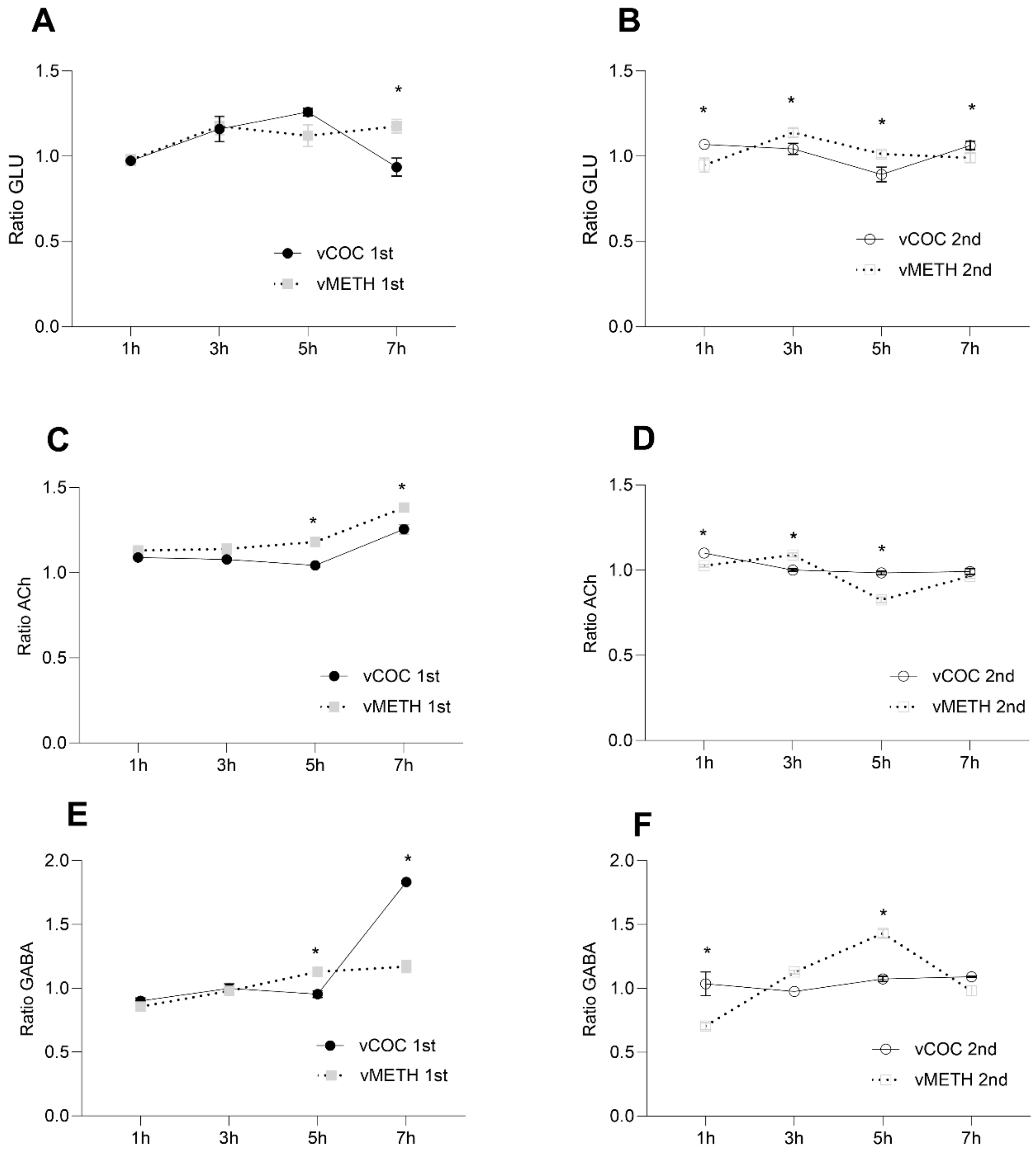

Figure S3. Temporal dynamics of changes in the concentration ratio of glutamate, acetylcholine and GABA semi-quantified without standards after vCOC and vMETH administration. The ratio of the relative proportion of glutamine, acetylcholine and GABA was measured in the heads 1h, 3h, 5h and 6h after one dose of vCOC (75 µg) and one dose of vMETH (75 µg) administered at 9:00, and two doses of vCOC (2 × 75 µg) administered at 9:00 and 15:00 and vMETH (2 × 75 µg) administered at 9:00 and 19:00. Psychostimulants were administered using the FlyBong method. Presentation of changes in the concentration ratio of glutamate **A)**, acetylcholine **C)** and GABA **E)** after exposure to one (vCOC 1st) and (vMETH 1st) dose. Two-way ANOVA with Tukey's multiple comparisons test. *: $p<0.05$. Representation of changes in the concentration ratio of glutamine **B)**, acetylcholine **D)** and GABA **F)** after exposure to two (vCOC 2nd) and two (vMETH 2nd) doses. Two-way ANOVA with Tukey's multiple comparisons test. *: $p<0.05$.
