# Supplementary material for: Distinct and Dynamic Changes in the Temporal Profiles of Neurotransmitters in Drosophila melanogaster Brain following Volatilized Cocaine or Methamphetamine Administrations
Source: Pharmaceuticals (Basel). 2023 Oct 19;16(10):1489. doi: 10.3390/ph16101489 (PMC10609923; doi:10.3390/ph16101489)
Supplement: Supplementary file 1 [file pharmaceuticals-16-01489-s001.zip › Table S1,S2.pdf]

Supplementary material

Table S1. MS/MS standards optimization parameters. The table lists, for each standard, the recording mode (polarity), precursor ion (*m/z*), fragmentor, fragment ions (*m/z*), collision energy (CE), retention time (t_R), slope (a) and section on the x-axis (b), R2, linearity range (linearity), detection limit (LOD), and quantification limit (LOQ).

**Standard**	**Polarity**	**Precursor (*m/z*)**	**Fragmentor (V)**	**Product ions (*m/z*)**	**CE (V)**	**t_R (min)**	**Y=ax + b** Slope (a)	Intercept (b)	**R^2**	**Linearity range (µg/mL)**	**LOD (µg/mL)**	**LOQ (µg/mL)**
Dopamin	+	153.9	60	118.6	10	1.0	37355.512266	-49.952304	0.9947	0.01-1	0.00441	0.01337
				90.9	20							
Octopamin	+	153.9	60	136.9	4	0.5	109396.977405	10.295928	0.9992	0.0001-1	0.000311	0.000941
				90.8	24							
Tyramin	+	137.9	60	120.9	4	1.5	167336.717374	79.131000	0.9975	0.001-1	0.001561	0.004729
				90.9	22							
				77.0	30							

*Quantifier ions are underlined.

Table S2. MS/MS parameters for components semiquantified without standards: acethlycholine (ACh), gamma-aminobutyric acid (GABA) and glutamate, N-acetyl dopamine, N-acethyl tyramine, cocaine (COC) and methamphetamine (METH). The table list the recording mode (polarity), precursor ion (*m/z*), fragmentor, fragment ions (*m/z*), collision energy (CE), and retention time (t_R).

**Compound**	**Polarity**	**Precursor (*m/z*)**	**Fragmentor (V)**	**Product ions (*m/z*)**	**CE (V)**	**t_R (min)**
ACh	+	147.0	135	<u>88.0</u>	25	0.6
				87.0	25	
GABA	+	103.9	135	69.0	5	0.6
				<u>45.0</u>	25	
				43.0	25	
Glutamate	+	147.9	135	129.0	25	0.7
				101.0	25	
				<u>84.0</u>	25	
N-acetyl dopamine	+	196.0	135	154.0	15	4.6
				137.0	15	
				119.0	30	
				91.0	30	
METH	+	150.5	135	<u>91.2</u>	20	5.2
N-acetyl tyramine	+	180.0	135	138.0	30	5.3
				121.0	30	
				91.0	40	
				<u>77.0</u>	40	

COC	+	304.1	100	182.1	30	7.2

*Quantifier ions are underlined.
